# Recent Advances in the Excipients Used for Modified Ocular Drug Delivery

**DOI:** 10.3390/ma14154290

**Published:** 2021-07-31

**Authors:** Melitini Koutsoviti, Angeliki Siamidi, Panagoula Pavlou, Marilena Vlachou

**Affiliations:** 1 Department of Pharmacy, Division of Pharmaceutical Technology, School of Health Sciences, National and Kapodistrian University of Athens, 15784 Athens, Greece; melinakoutsoviti@ymail.com (M.K.); asiamidi@pharm.uoa.gr (A.S.); 2 Department of Biomedical Sciences, Division of Aesthetics and Cosmetic Science, University of West Attica, 28 Ag. Spyridonos Str., 12243 Egaleo, Greece; ppavlou@uniwa.gr

**Keywords:** ocular drug delivery, eye, excipients, chitosan, hyaluronic acid, poloxamer, PLGA, PVCL-PVA-PEG, cetalkonium chloride, gelatin

## Abstract

In ocular drug delivery, maintaining an efficient concentration of the drug in the target area for a sufficient period of time is a challenging task. There is a pressing need for the development of effective strategies for drug delivery to the eye using recent advances in material sciences and novel approaches to drug delivery. This review summarizes the important aspects of ocular drug delivery and the factors affecting drug absorption in the eye including encapsulating excipients (chitosan, hyaluronic acid, poloxamer, PLGA, PVCL-PVA-PEG, cetalkonium chloride, and gelatin) for modified drug delivery.

## 1. Introduction

To date, ocular drug delivery has been limited to topical applications, intraocular/periocular injections, or systemic administration. Although topical drops are the most convenient mode of ocular drug delivery, maintaining an effective concentration of the drug in the target area for a sufficient period of time is a challenging task [1]. Therefore, there is a need for the development of effective and modified drug delivery strategies to the eye. This could be achieved either by incorporating new excipients into the existing formulations or by novel approaches to drug delivery. In this article, we discuss the limitations of conventional ocular therapy and review recent advances in materials science and drug delivery approaches used to improve ocular bioavailability.

## 2. Anatomy and Physiology of the Eye

The eyes are one of the most versatile sensory organs of the human body, with the significant role of converting images into electrical signals [2]. The eye occupies about 20% of the orbital volume. It is spherical in shape and is located in the orbital cavity. Ligaments and muscles are responsible for holding the eyeball within the cavity [3,4,5]. The human eye is composed of three layers: the fibrous tunic, the uvea, and the retina [6].

The fibrous tunic (outer fibrous layer) includes the cornea and sclera. The cornea is considered the most delicate tissue. It is a thin, diaphanous structure that contains no vessels. Its main function is refraction of light, which occurs due to its spherical shape, reflective index, and flat surface [3,4]. The sclera is referred to as the “white of the eye” [2]. It is a firm, elastic structure composed of collagen fibers whose function is to maintain intraocular pressure [7]. 

**Figure 1:**
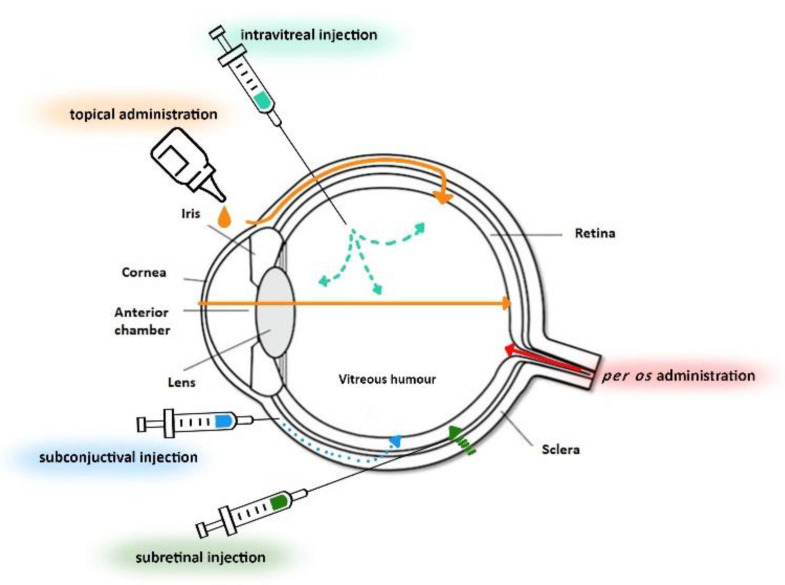
Basic anatomy of the eye and methods/routes of ocular drug administration: topical administration, subconjunctival injection, subretinal injection, intravitreal injection, and *per os* administration.

The uvea (middle layer) is characterized by a high blood supply and consists of the iris, ciliary body, and choroid. The iris is located behind the cornea and is the colored part of the eye. This membrane has an annular opening in front of the lens called the pupil. The pupil can adjust according to brightness to allow a certain amount of light to fall on the retina. The ciliary body rests on the iris. Its main function is to produce aqueous humor, while also regulating the shape of the lens. The choroid, located at the back of the uvea, is a membrane responsible for nourishing and oxygenating the iris and retinal photoreceptors [2,4]. The most internal layer of the eye is the retina. It is a transparent structure consisting of many layers in which photoreceptors are located [7,8]. Furthermore, the aqueous humor and vitreous humor are of great importance. The aqueous humor is a transparent fluid located in the anterior and posterior cavity of the eye, while the vitreous humor is a transparent jelly-like fluid located in the posterior part of the eye. Its role is to nourish the cornea or hold the eyeball in place [2,4]. Another important structure is the lens, an elastic organ that allows light to reach the retina [3,4]. Figure 1 depicts the basic anatomy of the eye, showing the exterior and interior of the eye including the sclera, cornea, iris, lens, retina, anterior chamber, vitreous humor, and drug flow in the ocular area.

## 3. Ocular Drug Delivery

The high sensitivity of the eye, as well as ocular biological barriers, have attracted the interest of researchers as they present difficulties in drug delivery [9]. Ocular diseases are very common and plentiful, exceeding 100 different types [10]. Some of the most common eye diseases are dry eye syndrome (DES), cataract, glaucoma, uveitis, conjunctivitis, age-related macular degeneration (AMD), diabetic macular edema (DME), and diabetic retinopathy (DR). There is a wide range of medications administered topically or intraocularly for these conditions, such as antibacterials, antivirals, anti-inflammatories, antifungals, cycloplegics, mydriatics, miotics, and local anesthetics [11,12,13,14]. Ocular drug formulations are divided into liquids (solutions, suspensions, and emulsions), semiliquids (ointments and gels), and solids (ocular inserts, contact lenses, microneedles, minidiscs, collagen shields, minitablets, sprays, and ocular iontophoresis). In addition, there are intraocular delivery systems such as injections and implants [15,16,17]. 

The administration of ocular drugs is mainly topical, but there are also periocular and intraocular administrations [18]. The topical route of administration is preferred over the systemic route due to several advantages. First of all, this route is considered safer with fewer side effects compared with the systemic route of administration. This is because the drug is administered directly into the eye lobe, thus protecting the rest of the body from systemic exposure. Agarwal et al. refer to this route as the only way to achieve therapeutic concentrations in the eye [19]. Moreover, topically applied drugs escape first-pass metabolism and thus reach higher concentrations in the target area [20]. From the patient’s perspective, topical administration is more convenient. Because it is noninvasive, topical formulations are more manageable and result in better patient compliance. Notably, instillation is an uncomplicated and painless method of administering medication to the anterior segment of the eye [21,22].

## 4. Factors That Affect Drug Absorption in the Eye 

Drug delivery via the eye is one of the most challenging areas of research in the pharmaceutical sciences due to the eye’s special and unique anatomical and physiological barriers. These barriers are of great importance as they affect the drug concentration and bioavailability, and hence the pharmacological effect of ocular drugs [23]. In particular, the percentage of drug reaching the target tissue via the topical route of administration is estimated to be less than 5%, while most of the ocular formulations on the market are topical applications due to the difficulty in overcoming ocular barriers [24]. 

The first main barrier is the tear film. This is secreted by the lacrimal gland and consists of three layers: the lipid layer, the aqueous layer, and the mucosa, while underneath the mucosa are the cornea and conjunctiva. Considering that the tear film is in direct contact with the environment, it provides a high level of protection to the eye [25]. The estimated volume of most ocular formulations is 35–56 μL, more than the 30 μL volume that a healthy eye can hold. This difference in fluid volume provokes the blink reflex. Another important factor is the high turnover rate, which leads to minimization of contact time between drug and ocular tissue. However, this rate also depends on the properties of the drug. When the drug is applied to the ocular tissue, it mixes with the tear fluid and flows into the nasolacrimal duct. As a result, the contact time with the ocular tissue is limited and therefore the effective concentration of the drug is low [26,27]. 

In addition to the tear film, another significant factor in drug elimination is the aqueous humor. The ciliary body secretes aqueous humor, which flows toward the cornea in the opposite direction to the drug pathway. The aqueous humor mechanism occurs via the chamber angle and Schlemm’s canal, as well as the venous blood flow of the anterior uvea. Both mechanisms prevent the passage of large hydrophilic molecules [2]. 

The conjunctiva is a hydrophilic tissue permeable to peptides and composed of mucus. Due to its strong vascularization and the existence of lymphatic vessels, this anatomical structure is considered ineffective as the drug mainly enters the systemic circulation [28].

The cornea, also located in the anterior chamber of the eye, is composed of epithelium, stroma, and endothelium. Epithelium is characterized by lipids and its cells are tightly interconnected, limiting the permeability of hydrophilic and ionized molecules. The stroma, on the other hand, is highly hydrophilic. Both epithelium and stroma are not permeable to macromolecules (above 50,000 kD). The endothelium, the inner layer on the cornea, is a lipophilic cell layer composed of phospholipids. Overall, the cornea is permeable to amphiphilic molecules that have both hydrophilic and lipophilic character in their structure [29].

Another barrier is the blood–eye barrier, which includes the blood–aqueous barrier (BAB) and the blood–retinal barrier (BRB). The BAB consists of the dense capillary iridal vascular endothelium and ciliary nonpigmented epithelium and regulates the movement of solvents between the anterior and posterior chambers of the eye. According to Jonghwa Lee and Ryan M. Pelis, BAB has many different drug transporters that appear to be involved in the limited exposure of the eye to drugs [30]. In addition, the flow of ions, proteins, and water into and out of the retina is controlled by the BRB. Normal vision is highly dependent on this specific structure that prevents macromolecules and harmful molecules from entering the retina. The BRB consists of two sections, the inner and the outer. Both have tight junctions and prevent paracellular movement from the blood to the retina and vice versa. The junctions of the outer section of the BRB are located between retinal epithelial cells (REC), which lie beneath Brunch’s membrane and are considered a substantial barrier. Due to their structure, hydrophilic and large-volume molecules are excluded from the transscleral pathway [31,32]. 

The posterior orbit is mainly covered with the resistant tissue of the sclera. Consisting of collagen fibers in a proteoglycan matrix, the sclera is an obstacle for lipophilic molecules. On the other hand, since the pores of the matrix are negatively charged, hydrophilic drugs are more permeable. An important aspect to consider in ocular drug delivery is that the sclera is a tissue whose permeability does not change with age [33,34]. However, the ability of a drug to cross the scleral barrier seems to depend on the physiochemical properties of the molecule, its charge, and its molecular radius [2]. 

P-glycoprotein and multidrug-resistant protein (MRP) are present in the apical or basolateral epithelial cells of ocular tissue. These two efflux pumps affect the bioavailability of drugs in the eye, as their main function is to channel unknowns into the ocular molecules. Specifically, P-glycoprotein is responsible for eliminating cationic molecules, while MRP is an anionic transporter. Both efflux pumps limit the entry of large and natural agents [27,35]. 

In addition to static and dynamic barriers, the eye exhibits metabolic barriers. Metabolic enzymes are present in all ocular tissues, with the ciliary body and retinal pigment epithelium having the highest metabolic activity. The major metabolic enzymes are CYTP450 and lysosomal enzymes. CYT450 is involved in the first phase of metabolism and its role is to create or expose polar groups in a molecule, promoting its excretion. The rest of the products, which include the enzymes acetyltransferase, sulfotransferase, and glutathione S-transferase, continue polarization in phase two. Lysosomal enzymes are found in the lysosomes and melanosomes. These enzymes act under acidic environments and catabolize/degrade proteins, polysaccharides, nucleic acids, and lipids [36]. 

Finally, scientists believe that the shape of the eye itself is an obstacle to ocular drug delivery [37].

## 5. Pharmaceutical Dosage Forms for Ocular Administration

An appropriate ocular formulation should overcome the multiple barriers of the ocular tissues and release the drug at the target site without causing tissue damage. Specifically, the optimal ocular drug delivery system must achieve constant concentrations at the target site at a given time, must be nontoxic, and must be easy to use [29]. The field of developing new ophthalmic formulations is highly challenging. Several new formulations are available but topical eye drop solutions and emulsions are still predominant because they are easy to prepare, act immediately, and are accepted by patients [19,38]. In addition to eye drops, there are other topical ophthalmic preparations such as emulsions [37], ointments, and gels [39,40]. There are also novel formulations such as ocular inserts and implants [41,42], injections [43], microneedles [17], nanowafers, punctum plugs [37], minitablets [15], and sprays [44]. Iontophoresis is another popular approach [45]. 

As previously mentioned, eye drops are the most commonly used formulation for topical treatment of ophthalmic diseases. According to the International Pharmacopoeia, eye drops can be solutions, suspensions, or emulsions in an aqueous or oily medium [46]. Since they are administered directly into the conjunctival sac, they contain at least one preservative to ensure sterilization, antioxidants to prevent oxidation, and buffers and isotonic modifiers to achieve a pH of 7.4 (compatible with the pH of the tear film) [47]. In the case of suspensions/emulsions, fatty acids, waxes, and suspending/emulsifying agents are also used. When these solids are formulated into foams/sprays, a propellant mixture is added. A major advantage of eye drops is that the active ingredient is already solubilized, resulting in faster response [1]. However, eye drops also have significant disadvantages. The residence time of the drug at the ocular surface is limited due to the various ocular barriers, such as nasolacrimal drainage, tear blinking, and tear flow, resulting in poor bioavailability. This results in the need for frequent administration, leading to patient compliance issues. In addition, the additives in the formulation can lead to potential toxicity of the delicate ocular tissues [48,49]. A critical aspect is that the eye drops are only effective in treating anterior eye disease [50].

These serious drawbacks prompted scientists to come up with new methods to deliver drugs to the ocular tissues. A common approach is the use of semisolid systems, with the aim of delaying drug clearance and increasing bioavailability by increasing viscosity [51]. Semisolids usually consist of a preservative and a vector, while in gels a gelling agent and a humectant are added. In creams and ointments, oily or aqueous bases and emulsifiers are added. Gels are colloidal systems consisting of a liquid medium (continuous phase) in which microscopic particles of 1–1000 nm are dispersed to form the dispersed phase [52]. Polymeric gels can be classified into two groups: classically designed gels and in-situ forming gels [39]. The first subgroup refers to simple viscous solutions that remain the same after administration without any change. In contrast, in-situ gelling systems are viscous fluids that transform in situ from solution form to gel form in response to an environmental stimulus to which they are exposed. Specifically, this gelling occurs through physical or chemical crosslinking of the polymer chains. Physical mechanisms include swelling, in which the gel absorbs water from its environment and expands, and diffusion, which refers to the diffusion of the polymer solution solvent into adjacent tissues, resulting in a solidified polymer matrix. In addition, chemical crosslinking involves chemical reactions of precipitation, the involvement of enzymes, and the generation of signals through photoinitiated polymer reactions [53]. The polymers used can be natural or semisynthetic and are classified into thermosensitive, pH-sensitive and ion-sensitive polymers, depending on the stimulus. In particular, thermosensitive polymers are characterized by a lower and upper critical solution temperature (LCST and UCST, respectively) at which the phase transition occurs. Thus, the polymer is liquid at temperatures between 20–25 °C and, at temperatures above 33.8 °C, the phase changes. For pH-relative polymers, the ability to change phase depends on the ionizable groups they contain, while ion-activated polymers interact with cations in the tear fluid, resulting in an increase in viscosity [40]. 

One alternative formulation used for controlled delivery to the eye is ocular inserts. This type of formulation consists of multiple layers saturated with the drug. Inserts are predominantly classified based on their solubility into insoluble, soluble, and bioerodible inserts [54]. Insoluble inserts are composed of nonbioerodible polymers and the target area (upper or lower fornix or cornea) is determined by the shape and size of the device. They are generally preferred because of several advantages. Some of these include improved effect on the target area, a lack of need for frequent dosing, and better control over release rate and dose [55,56]. Researchers divide insoluble inserts into three groups: membrane-guided reservoir inserts, minidiscs, and soft contact lenses. Starting with reservoirs (also called ocuserts), the leading mechanism is diffusion. In the center of the insert is a solid reservoir that contains the medication and is covered with membranes. The selection of a suitable membrane is of great importance as the rate of release of the drug is controlled by diffusion through it. Minidiscs function by osmosis and can be divided into two types. The first consists of two parts: a central matrix containing the drug and an osmotic solution surrounded by polymers. In the second, the active ingredient and the osmotic solution are arranged separately, both surrounded by suitable membranes. The system is covered with a film that allows the penetration of tear fluid, saturating it and leading to its dissolution. The third category of insoluble inserts is soft contact lenses. They are three-dimensional structures consisting of hydrophilic or lipophilic polymers. When the matrix is placed in the drug solution, it absorbs the drug and later delivers it to the target area at a nonstable rate [57]. On the other hand, soluble inserts dissolve or erode so that they do not need to be removed at the end of the release. The main categories are soluble ophthalmic drug inserts (SODI), bioadhesive ophthalmic drug inserts (BODI), and collagen shields, based on the type of polymer used. These structures undergo various processes to absorb and then release the drug with the cooperation of tears. Tears penetrate into the insert and contribute to the formation of a gel layer around the core. Therefore, the release rate is still under control [41]. Finally, the coat polymer of the active ingredients marks an ophthalmic system as bioerodible or not. The release of the drug from bioerodible inserts occurs due to bioerosion of the matrix [57]. Excipients used in inserts or films are emollients (usually polymers) that form the delivery system, humectants, and solvents.

## 6. Excipients Used in Ocular Formulations for Modified Drug Delivery

### 6.1. Chitosan

Chitosan is a natural unbranched polysaccharide with a straight chain of randomly distributed monosaccharides, built up from *N*-acetyl-*D*-glucosamine and *D*-glucosamine with *β*-(1-4) linkage. Introduced by Rouget in the mid-19th century, chitosan has recently attracted the attention of the scientific community due to its uniqueness and versatility [58,59]. The term chitosan does not refer to a specific molecule but instead to a group of polymers derived from the deacetylation of chitin—the second-best known biopolymer after cellulose—which is derived from various marine species of crustaceans and cephalopods. The conversion of chitin to chitosan can be done by enzymatic or chemical deacetylation [60]. 

The chitosan obtained is characterized by a wide range of degrees of deacetylation and molecular weights, which determine the quality and properties of the polymer. Solubility strongly depends on these characteristics of the chitosan. Specifically, chitosan derivatives with an average degree of deacetylation higher than 50% are soluble only in acidic solution but show no solubility in alkaline solutions or those at neutral pH. Moreover, the chitosan structure contains hydroxyl and amino groups. In an acidic medium, the molecule becomes polycationic and thus has the ability to interact with various negatively charged molecules and macromolecules [61]. The interaction between positively charged chitosan and the negatively charged mucosal surface enables the polysaccharide to adhere to this surface in a manner that confers mucoadhesive properties to the polysaccharide, making chitosan suitable for drug delivery to the mucosa [62,63]. Moreover, chitosan has been shown to be biocompatible and biodegradable while not appearing to cause toxicity. When it enters the human body, chitosan is mainly recognized and metabolized by lysozymes, producing harmless oligosaccharides. These three properties make chitosan a suitable polysaccharide for pharmaceutical applications [64]. In addition, chitosan has shown a variety of biological activities. It has high antimicrobial activity and killing rate while exhibiting low toxicity to human cells. It also has the ability to deliver hydrogen, resulting in antioxidant activity. Finally, chitosan limits the growth of cancer cells by increasing the amount of cytolytic T lymphocytes [60].

Scientists have also formulated solutions with glucocorticoids; notably, they have succeeded in creating a water-insoluble methyl-β-cyclodextrin-ammonium chitosan system that exhibited mucoadhesiveness and cytocompatibility [65]. Piras, with another group of scientists, also created a methyl-β-cyclodextrin-ammonium-chitosan system, but this time using nanoparticles. The results showed decreased mucoadhesiveness compared with the first experiment, but improved water-assisted transport through the mucus [66]. In addition, another group of researchers combined chitosan oligosaccharide with valylvaline and stearic acid to obtain a system that demonstrated good biocompatibility as well as mucoadhesive and penetrating properties [67].

An ophthalmic vehicle composed of chitosan, guar gum, and cellulose derivatives was also developed. Using a lubricant as API, the final system was characterized by mucoadhesiveness, improved rheology, and good physicochemical properties. Moreover, the preparation was simple and stable for more than one month [68].

Chitosan was also used in nanoscale formulations. Researchers attempted to improve the ocular delivery of dorzolamide hydrochloride by preparing nanoparticles composed of polycaprolactone, polyvinyl alcohol, and chitosan. The resulting nanoparticles exhibited biphasic behavior, delayed release, and ability to penetrate the cornea while causing no irritation [69]. Another group of scientists prepared conjugates of chitosan and glycol and the active ingredient dexamethazone. These conjugates were able to self-assemble and form nanoparticles in aqueous environments. Compared with the aqueous solution, these nanoparticles showed improved precorneal retention and mucoadhesion [70]. 

In addition to the solutions, some researchers attempted to form a cationic nanoemulsion with ibuprofen as the active ingredient for the treatment of dry eye. Chitosan was used as the cationic agent, while the anionic agent was lecithin. The results showed great mucoadhesion and biocompatibility [71].

Chitosan also shows great potential as an excipient in hydrogels. Hydrogels are networks of crosslinked polymers that can have physicochemical properties that make them suitable for ocular drug delivery [72]. 

Researchers have used chitosan to formulate both in-situ and thermosensitive gels. Using different antibiotics as active ingredients, Ameeduzzfar et al. created a chitosan–polyvinyl alcohol–gelatin gum delivery system that showed enhanced bioavailability at the corneal epithelium and no visible irritation along with enhancement of antibacterial activity [73]. Moreover, researchers tried again to create an in-situ gel, this time using chitosan nanoparticles loaded with levofloxacin, with the same results [74]. In addition, Song et al. and Kong et al. formulated thermosensitive gels. The first group of researchers formed a hydrogel from chitosan and gelatin crosslinked with *β*-cyclodextrins and genipin, while the second group assembled a gel composed of chitosan, carboxymethyl chitosan, and glycerophosphate. A beta-blocker and a fluoroquinolone were used as the active ingredients, respectively. The results of both groups were similar, emphasizing the prolonged contact time which ensured an even and sustained release of the active ingredient [75,76]. In addition, a thermosensitive chitosan–gelatin-based hydrogel containing curcumin-loaded nanoparticles and latanoprost was formulated as a dual-drug delivery system for glaucoma treatment [77]. The results indicated that using this newly developed system, both active ingredients displayed a sustained-release profile.

Finally, some other researchers used chitosan to design polymeric films and inserts. A chitosan/poly(2-ethyl-2-oxazoline) film with fluorescein sodium salt was prepared, resulting in a formulation with great biocompatibility and improved retention time [78]. The ocular insert was prepared to study the sterilization and pharmacokinetics of chitosan. The formulation showed good retention in the eye as well as good pharmacokinetics, considering that degradation started more than 12 h after ocular application [79].

### 6.2. Hyaluronic Acid

Hyaluronan (hyaluronic acid, HA) is a natural linear polymer composed of two types of sugars: the amino sugar *N*-acetyl-d-glucosamine and the uronic sugar d-glucuronic acid, linked by *β*-1,4-glycosidic bonds. This biopolymer is part of a large group of polysaccharides called glycosaminoglycans (GAGs). The different molecules belonging to the family of GAGs have similar structure and molecular weight and are synthesized in Golgi bodies and endoplasmic reticulum [80]. However, in terms of most of the above, HA differs from other GAGs. Specifically, hyaluronan is not synthesized in the Golgi but in the plasma membrane by synthases, while it has a higher molecular weight compared with other members of the group. The difference in molecular weight seems to play a crucial role in the properties of hyaluronan, such as viscosity, which progressively increases with molecular weight [80,81].

Regarding its chemical profile, HA is anionic at physiological pH, due to the carboxylate groups of the molecule, and, when balanced with cations, it forms salts called hyaluronans. This charge determines the chain structure and interactions with neighboring tissues, thereby affecting the water solubility and rheological properties of the polymer [82]. 

First isolated from the vitreous humor of cows by Mayer and Palmer in 1934, HA is a polymer that occurs naturally in various tissues of the human body [83]. It is found both extracellularly and intracellularly, but mainly in the extracellular matrix. Half of the hyaluronic acid in the human body is found in the intracellular space of skin cells. Its role is of great importance because it not only forms a matrix for the cells, but also changes the volume of the skin. In addition, the formation of new cells and the repair of skin tissue are closely related to the function of HA. Skin tissue is the main barrier that protects internal tissues from the unfriendly external environment. Hyaluronan contributes to this by preventing free radicals from sunlight from reaching the skin tissue, thus forming a protective shield. In addition, this biopolymer is found in synovial fluid where it lubricates the joint and absorbs vibrations [84]. Additionally, HA exists in various structures of the eye—mainly as a lubricant. Consequently, it is a biocompatible polymer that can be degraded enzymatically or chemically [85]. For all these reasons, as well as the fact that the polymer has some groups that are suitable for modification, HA is a promising excipient that can contribute to the controlled and targeted release of drugs [86]. 

Scientists designed a thermosensitive in-situ hydrogel composed of poly(*N*-isopropylacrylamide) and HA. Using ketoconazole as the active ingredient, the scientists succeeded in enhancing the therapeutic effect and minimizing toxic effects in in vivo studies [87]. 

Moreover, some researchers from China formulated a glycol–chitosan oxidized HA film loaded with levofloxacin and dexamethazone. The results of the study showed the potential of the formulation to differentiate the releases of two drugs (immediate release of the first drug and a delayed release of the second) while maintaining biocompatibility of the formulation. The delivery system also showed anti-inflammatory activity [88]. Hyaluronic acid was again used as an excipient in the formulation of an ocular film, but this time it was crosslinked with itaconic acid and loaded with acetazolamidehydroxypropyl-*β*-cyclodextrin-triethanolamine complexes. The presence of HA resulted in better bioadhesion and biocompatibility, along with the possibility of removing the need for preservatives [89].

Hyaluronic acid has also been used in the formulation of nanoscale delivery systems. A group of scientists designed lipid–polymer hybrid nanoparticles modified with hyaluronic acid and loaded with moxifloxacin hydrochloride. The formulation showed great bioavailability, partly due to the modified hyaluronic acid in the surface of the nanoparticles triggering endocytosis [90]. Additionally, researchers used hyaluronic acid in combination with glycosaminoglycan as a stabilizer of acetazolamide nanoparticles. The final spray-dried nanosuspension showed stability and maintenance of dispersion for up to 6 months [91].

### 6.3. Poloxamer 

Poloxamers are ABA-type nonionic copolymers consisting of two distinct units, polyoxyethylene and polyoxypropylene. Due to the nature of these two units, which are hydrophilic and hydrophobic, respectively, poloxamers are referred to as amphiphilic [92]. Poloxamers are commonly used as surfactants in the formation of in-situ gels. They come in the form of liquids, pastes, or flakes and are commercially available under various brand names such as Pluronic, Synperonic, and Tetronic. The synthesis of these copolymers is based on the sequential addition of the two different block types (methyl oxide and epoxide) to propylene glycol—an organic compound with low molecular weight and water solubility. This synthesis is carried out in the presence of a catalyst, such as sodium or potassium hydroxide [93]. 

The distinction between the different poloxamers lies in the length of the lipophilic and hydrophilic units, which determine the properties of the copolymer. The best-known types are poloxamers 407 and 188, whose names indicate the molecular weight of their methyl oxide and epoxide groups, respectively [94]. Due to the presence of both hydrophilic and hydrophobic groups, poloxamers are characterized as thermoreactive and change their physical properties as a function of temperature. This reversible phenomenon, called gelation, refers to the transformation of the poloxamer from a liquid to a semisolid state and is dependent on the sol-gel temperature. Temperatures above the sol-gel temperature result in the gel form, while temperatures below it maintain the aqueous solution form of the poloxamer. This conversion from one form to the other depends on the existence of the hydrophobic units interacting with each other. An increase in temperature beyond the sol-gel temperature results in a corresponding increase in micelle density, leading to gel formation. This transition has an impact on the rheology and viscosity of the poloxamer [95,96].

Regarding their stability, poloxamers have been shown to degrade through the mechanisms of autooxidation and chain scission involving free radicals [97].

In ophthalmology, the use of poloxamer 407 raises many issues that need to be overcome. First, the sol-gel temperature seems to be unsuitable, ranging between 20 and 25 °C. Moreover, the concentration needed to achieve gelation is high, which affects osmolarity [98]. Nevertheless, they are considered important excipients as they can be used for targeted drug release [99]. For these reasons, poloxamer 407 is usually combined with poloxamer 188 to optimize the temperature range and expand its use in drug formulation [98,100].

Researchers have used poloxamer 407 in combination with vitamin E to form a cyclosporine solution. In this way, the solubility and stability of the drug were increased. Moreover, the delivery system exhibited a reservoir effect, while no irritation occurred [99]. Another group of researchers formed an in-situ gel of moxifloxacin hydrochloride by combining poloxamer 407, gellan gum, and carbopol. This study showed the potential of this formulation, which is characterized by great strength and no irritation [101].

Many studies also deal with the combination of poloxamer 407 and poloxamer 188. Researchers have formulated an in-situ gel with the two types of poloxamer and carbomethylcellulose [102], while other researchers prepared a nanohydrogel using the two polymers in combination with soy lecithin E200, lecithin oil, glycerol, and polycarbophil [103], while yet another group used poloxamers 407 and 188 and chitosan gel [104]. All three studies showed sustained release of the drug.

### 6.4. PLGA

PLGA is a linear synthetic biopolymer composed of the monomers polylactic and polyglycolic acid. Polylactic acid has two enantiomorphs due to the presence of an asymmetric carbon: d-polylactic acid (PDLA) and *L*-polylactic acid (PLLA), which are commonly used in PLGA in a 50:50 ratio [105]. 

In terms of its properties, PLGA is soluble in various solvents and can be formed into a wide range of shapes and sizes. However, not all PLGA types exhibit the same properties as they are mainly influenced by the molecular mass and the ratio of the two monomers. Polylactic acid is found in both crystalline and amorphous forms, while polyglycolic acid is crystalline due to its methyl group. PLGA, on the other hand, is found in amorphous form. Moreover, PLGAs with a high content of polylactic acid are more hydrophobic due to the presence of methyl groups. This hydrophobicity has as a consequence a limited water uptake and a slower degradation rate [106].

As mentioned earlier, PLGA is a biopolymer which means that it degrades into naturally occurring, nontoxic products. The degradation process can be divided into four main phases: hydration, initial degradation, constant degradation, and solubilization. The final byproducts are lactic acid and glycolic acid [107]. There are several factors that affect the rate of degradation; these can be material, processing, or physiological factors. Some of them are molecular weight of polymer, glass transition temperature, and drug-loading properties such as drug size and hydrophilicity [108]. 

Researchers have used PLGA with other excipients to form nanoparticles that could be used for the treatment of various ocular inflammations. These nanoparticles were characterized by tolerability and permeability through the cornea, and showed great therapeutic effects [109]. Another group of scientists created lipid polymer nanoparticles with a PLGA nanocore loaded with brinzolamide to achieve sustained release of the drug, better permeation through the cornea, and improved therapeutic effect [110]. 

However, PLGA has limitations that need to be overcome. A promising way to formulate better delivery systems is to incorporate other types of copolymers, such as PEG [111]. 

Two different research groups from China have developed PLGA–PEG–PLGA controlled release injections into the conjunctiva. The first group prepared a double controlled release system with insulin-loaded nanoparticles/PLGA–PEG–PLGA hydrogels (ICNPH) [112], while the second group prepared a thermogel with rhodamine B and coumarin 6 as labeling agents [113]. Both formulations were found to be safe and biocompatible.

### 6.5. PVCL–PVA–PEG

In recent years, a novel block copolymer consisting of polyvinylcaprolactam–polyvinyl acetate–polyethylene glycol (PVCL–PVA–PEG) has attracted the attention of scientists. This polymer combination, marketed under the trade name Soluplus, is an amphiphilic polymer with a molecular weight of 90 to 140 kDa which is soluble in both water and organic solvents [114]. As an amphiphilic molecule, it has many advantages. First, its chains can orient themselves to give the molecule a structure suitable for the formulation of solid dispersions and solutions [115]. It is also proposed to increase the solubility and bioavailability of poorly soluble molecules through the formed micelles. Moreover, as a polymer, it has a tendency not to absorb moisture from the air [116,117].

The researchers prepared PVCL–PVA–PEG micelles loaded with myricetin. The results were very promising as the system caused no irritation or toxicity and improved permeation, while the drug was more soluble and stable and showed increased anti-inflammatory activity [118].

### 6.6. Cetalkonium Chloride 

Cetalkonium chloride (CKC) is a quaternary ammonium compound of the Al-cyl-benzyl dimethylammonium chloride family with antiseptic properties. It is mainly used topically for eye and mouth infections and its mechanism of action is based on its positive charge [119]. However, CKC is not only used as an active ingredient but also has much potential as an adjuvant. The different molecules of the alkylbenzyldimethyl ammonium chloride family differ in the length of the alkyl chain, which affects their solubility in different solvents. CKC, with 16C in the chain, exhibits high lipophilicity and shows limited solubility in water [120].

This excipient has been mainly used in the formulation of ocular nanoemulsions. The high lipophilicity leads to its distribution in the nonaqueous phase of an oil-in-water emulsion. This results in a high zeta potential at the surface of the nanodroplets, while no free molecules can disperse in the aqueous phase, minimizing ocular toxicity. In addition, CKC has no preservative effect and is considered safe for ocular use [121,122,123].

Researchers prepared a poly-2-hydroxyethylmethacrylate hydrogel that contained an oil-in-water microemulsion. The oily phase of the microemulsion consisted of ethyl butyrate, while the water phase was composed of two nonionic surfactants and the cationic surfactant CKC. The formulated contact lenses achieved prolonged residence time of the active ingredient [124]. In addition, another group of researchers investigated the effect of CKC and sterylamine in vitamin E-loaded lenses. It was found that the cationic surfactants increased the amount of drug that could be loaded, while the system showed delayed release [125].

### 6.7. Gelatin

The breaking of crosslinks of the polypeptide chains of collagen, which is accompanied by the disruption of peptide bonds, leads to the formation of the polypeptide gelatin [126]. The amino acid sequence of gelatin differs depending on the source of collagen. The source material and the conditions under which the collagen is converted into gelatin—such as pH, extraction time, and temperature—also determine the molecular mass of its chains. If the collagen is pretreated with an acidic solution, the amino groups are not affected, resulting in type A gelatin. On the other hand, if the collagen is pretreated with an alkaline solution, asparagine and glutamate become aspartate and glutamate due to the hydrolysis that takes place, and the finished gelatin is type B. The main sources of gelatin are animal connective tissues such as skin and bone [127]. 

In terms of its properties, gelatin exhibits high dissolution in water, which makes it an unsuitable carrier for long-term drug delivery systems. Moreover, it exhibits a low rate of degradation in vivo. This rate can be improved and regulated by changing the crosslinking density of the carrier by increasing the reaction time or increasing the concentrations. In addition, the crosslinking of the polypeptide has an impact on other properties of gelatin, such as its thermal and mechanical stability and the possibility of hydration [128]. In addition, gelatin has much potential in the pharmaceutical industry due to its gelling, emulsifying and foaming properties, and thus is capable of forming thermoreversible gels. When discussing gelatin, a Bloom index is usually used. The Bloom value or gel strength ranges from low (below 150) to high (200–300) and indicates the quality of the gel in terms of its gelation and viscoelasticity [129]. Gelatin has many other advantages that make it suitable for the formulation of drug delivery systems. First of all, it is widely available and not expensive. Moreover, it is not as antigenic as collagen, while some amino sequences can help to improve cell biological behavior. Finally, gelatin has various groups on its molecule that allow chemical modification [127].

Some researchers have investigated gelatin A as a potential excipient in drug delivery systems for timolol maleate. In an initial study, liposomes with a gelatinized core were developed and found to be safe, biocompatible with ocular tissues, stable, and achieved sustained release of the drug [130]. Other researchers created gelatin nanoparticles. Shokry Miral et al. used gelatin A for the nanoparticles and achieved limitation of edema, which usually occurs after timolol maleate application [131]. On the other hand, a group of scientists formed gelatin B nanoparticles and also created a hybrid system with HPMC. The nanoparticles had the same antihypertensive effect as the marketed formulations, while the hybrid system showed a reduction in intraocular pressure, an extension of the effect, and a reduction in the duration to maximum effect [132].

**Figure 2:**
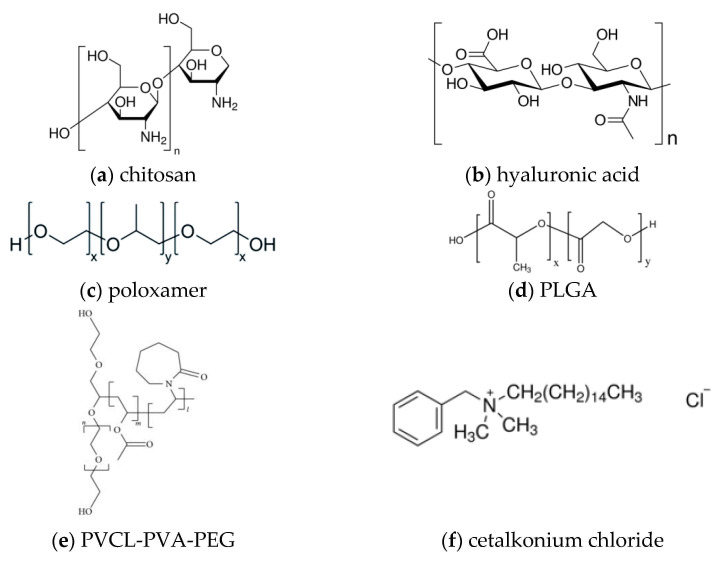

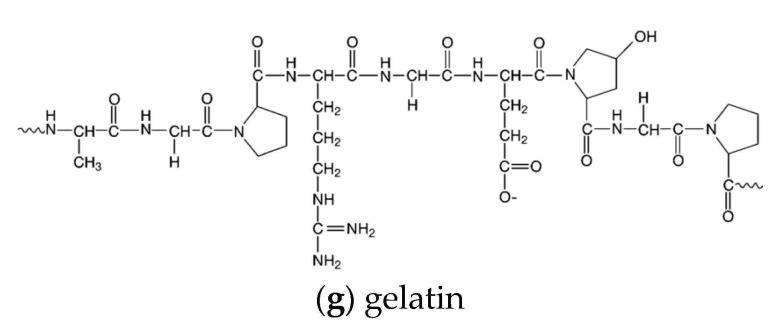
Chemical structures of: (**a**) chitosan, (**b**) hyaluronic acid, (**c**) poloxamer, (**d**) PLGA, (**e**) PVCL–PVA–PEG, (**f**) cetalkonium chloride, (**g**) gelatin.

Figure 2 depicts the chemical structures of the excipients mentioned above.

### 6.8. Pharmaceutical Nanotechnology in Ocular Formulations

Pharmaceutical nanotechnology has made a resurgence in research on eye disease treatment by presenting many advantages for ocular medications, including drug targeting, sustainability, and increased bioavailability. It is certain that nanomedicine will bring the medical market up to date, as the development of nanoformulations for the eye may enhance drug penetration through ocular barriers, prolong drug retention time, increase permeability and bioavailability of the drug, reduce degradation of unstable drugs, while being well tolerated by patients compared with conventional medications [133]. Additionally, ocular nanomedicine can circumvent hydrophobic drug solubility issues in aqueous media and provide sustained drug release with reduced toxicity and improved efficacy. Furthermore, certain types of ocular nanomedicines can target specific tissues and cells [134]. 

In more detail, the use of liposomes, nanoparticles, micelles, and dendrimers can provide more effective ocular drug delivery, since the therapeutic index can be increased and the ocular barriers diminished [135]. Liposomes are lipidic vehicles composed of phospholipids, cholesterol, and polymers; they can load hydrophilic drugs in the inner aqueous core and lipophilic drugs in the lipid bilayers [136,137]. Nanoparticles are usually composed of biopolymers and can offer modified drug release by prolonging drug retention time and thereby increasing ocular drug bioavailability [138]. Dendrimers are highly ordered branched polymeric molecules; drugs can be entrapped within their core or linked to their surface. The drug-release pattern could be controlled by their architecture [139]. Micelles are lipid molecules that arrange themselves in a spherical vesicle, consisting of a hydrophilic (polar head groups) and a hydrophobic (hydrophobic chain) area where hydrophobic drugs could be entrapped [140]. The properties of the biomaterials used in these drug delivery nanosystems affect the final biopharmaceutical and pharmacokinetic profiles [141,142].

Phospholipids can form many kinds of assemblies, such as liposomes and micelles that can provide sustained release of the active substance for prolonged periods [143]. Their amphiphilic nature and excellent biocompatibility renders them suitable to be employed as excipients in medicinal formulations. More specifically, soybean phosphatidylcholine [92,144,145] and egg phosphatidylcholine [91] have been successfully used in modified release ocular formulations.

The following tables represent an overview of the excipients used in modified release liquid (Table 1) or semisolid ocular formulations (Table 2), and inserts/films (Table 3). Table 4 gives an overview of the nanomaterials-based excipients used in modified release ocular formulations, and, in Table 5, the key characteristics of excipients and their potential advantages/disadvantages are listed.

## 7. Triggered Release of Drugs and Their Potential Applications

Conventional ocular formulations are characterized by a typical drug distribution to the area administered. As a result, certain areas exhibit poor uptake, while side effects might appear. A potential solution seems to be triggered release. Researchers aim to develop drug delivery systems which respond to a stimuli that differentiate one tissue or environment from another. 

As mentioned earlier, “in situ gelling systems are viscous fluids that transform from the solution form to the gel form, in response to an environmental stimulus to which they are exposed”. The most studied type of in-situ gelling systems are likely those that are triggered by temperature. As is widely known, the ambient temperature (20–25 °C) differs from the physiological temperature, which is 35–37 °C. Researchers, based on this temperature difference, use polymers that can be converted from liquid to gel when the surrounding temperature increases. These polymers are characterized by a low critical solution temperature (LCST) and an upper critical solution temperature (UCST). The optimum temperature-triggered polymer solution should retain its liquid properties below the LCST and transform into gel at temperatures above the UCST [53]. Many scientists have prepared temperature sensitive in-situ gelling systems that exhibit sustained ocular drug release [76,88,163].

Another type of in-situ gelling system are those triggered by pH. These solutions mainly depend on the pH of lachrymal fluids, and when exposed to such, are converted into gels. To produce pH-sensitive drug delivery systems, certain polymers, such as carbopol and cellulose acetate phthalate, are used. These polymers are characterized by the presence of certain groups (basic or weakly acidic) which in a specific pH lead eventually to changes in the polymer’s conformation and swelling [53]. According to Wadetwar et al., who prepared a pH-sensitive in-situ gel loaded with Bimatoprost nanoparticles, the final results exhibited prolonged release of the active ingredient, as well as increased precorneal resident time [164].

The third type of in-situ gelling system are those that are ion triggered. When the ion-triggered polymer solution meets the ocular surface, it starts to create crosslinks with the cations that are present, leading to gelation [53].

## 8. Future Development Direction of Ocular Delivery 

Over recent decades, the field of ocular drug delivery systems has developed rapidly due to the multiple limitations present which cannot be easily overcome with traditional ophthalmic technology. These shortcomings, regarding low bioavailability and use of invasive methods, have led to the rise of technologies which seem promising. Nanotechnology has shown great potential in modifying and controlling drug delivery. Nanomicelles, nanoparticles, nanowafers, liposomes, dendrimers, and microneedles can be used to reach the target location and deliver the active ingredient while enhancing drug bioavailability [37,165]. However, these technologies need further improvement, as new concerns have arisen regarding their use. The most important of which include ocular toxicity, the availability of proper in vivo models, and new sterilization techniques, as well as their physical stability [166].

Moreover, it is worth mentioning that drug-laden contact lenses could be efficiently used in the treatment of ocular diseases of the posterior chamber [6], where conventional eye drops cannot reach sufficient drug concentration levels. This limitation could be overcome using drug-loaded contact lenses; with 50% to 70% bioavailability, they could provide the posterior chamber with a therapeutic dose, leading to therapeutic effects [6].

## 9. Conclusions

In conclusion, ocular drug delivery is a truly challenging field, mainly due to the complexity of the ocular tissues and the barriers present in the eye. However, great strides have been made in the field of delivery systems to improve available treatments. Researchers have not only focused on finding new excipients, but have also investigated excipients already in use that could potentially play a critical role in improving ocular drug delivery. Chitosan, hyaluronic acid, poloxamers, and gelatin are just a few of the excipients that appear to be very promising and could potentially abrogate the serious limitations in ocular drug delivery that have yet to be overcome.

## Figures and Tables

**Table 1:** An overview of the excipients used in modified release liquid ocular formulations (solutions, suspensions, and emulsions).

Release Rate/Mucoadhesive Properties *	API(s)	Excipients Used in the Formulation	Reference
maintenance of mucoadhesive properties	dexamethasone	QA-Ch60-MCD => water soluble CS derivative 2-diethylaminoethyl chloride, DMSO, 1, 6-hexamethylene diisocyanate, triethylamine	[65]
gradual release in the initial 4 h	dexamethasone	QA-Ch-MCD vs. NPs	[66]
sustained	dexamethazone	CS oligosaccharide-va-lylvaline-stearic acid (CSO-VV-SA) nanomicelles	[67]
increased mucoadhesive properties	lubricant	CS, hydroxypropyl guar gum, NaCl, Na_2_HPO_4_, BZC	[68]
sustained (up to 12 h)	dorzolamideHCl	CS, Polycaprolactone, PVA	[69]
quick initial following sustained (<48 h)	dexamethasone	Succinated Dex, Glycol CS, *N*-hydro-xysuccinimide, *N*-(3-dimethylaminopropyl)-*N*′-ethylcarbodii- mide hydrochloride	[70]
relevant reservoir effect	cyclosporine	poloxamer 407, *D*-α-Tocopheryl, PEG 1000, succinate (Kolliphor^®^ TPGS), kolliphor^®^ TPGS, Trifluoroacetic acid, vit E, vit E succinate	[99]
sustained	fluorometholone	PLGA, poloxamer 188 (P188), Transcutol P^®^	[109]
sustained	brinzolamide	PLGA—SPC, soybean phosphatidylcholine	[112]
sustained	myricetin	PVCL-PVA-PEG	[118]
sustained (over 150 h)	diclofenac sodium	ethyl butyrate, 2-hydroxyethyl methacrylate, ethylene glycol dimethacrylate, azobis-iso-butrylonitrile, Tween 80, Brij 97, CKC	[124]
sustained	timolol maleate	cholesterol, gelatin (A), soya bean phosphatidylcholine, glycerol, Na_2_HPO_4_, KH_2_PO_4_, NaCl	[130]
burst effect following sustained	timolol maleate	gelatin (A) bloom 300 glutaraldehyde, glycine	[131]
extended release (4 days)	timolol maleate	gelatin (B) glyoxal solution 40%, glycine, HPMC	[132]
controlled	betaxolol HCl	montmorillonite/CS 1-(4, 5-dimethylthiazol-2-yl)-3, 5-diphenylformazan	[146]
constant over time	riboflavin	CS HCl, Arginine L HCl PBS, soy lecithin/polysorbate 80, poloxamer 407	[147]
modified release for up to 24 h	prednisolone	PASP-CEA-CD, CD-modified thiolated poly(aspartic acid), *L*-aspartic acid, cysteamine	[148]
increased adherence time	neomycin B, kanamycin B	DNA block copolymers	[149]
prolonged	acerazolamide	Cremophore RH40, Span 60, Tween 80, Brij 35, *L*-alpha-phosphatidylcholine	[150]
sustained	econazole nitrate	*L*-Cysteine linked to 6-mercaptonicotinamide => attached to Eudragit^®^ L100-55DMSO/glycerol/thiourea/6-chloronicotinamide/H_2_O_2_/PG/PEG 400/cremphor EL/5,5′-dithiobis (2-nitrobenzoic acid)/1-ethyl-3-(3-dimethylaminopropyl) carbodiimide hydrochloride/N-hydroxysuccinimide/BZC/methoxyphenylazo-2-naphthol/Triton^®^ X 100	[151]

* Release rate as stated by the author(s). Abbreviations: BZC: benzalkonium chloride, CKC: cetalkonium chloride, CS: chitosan, DMSO: hydrochloridedimethyl sulfoxide, HA: hyaluronic acid, PEG: polyethylene glycol, PG: propylene glycol, PVA: polyvinyl alcohol.

**Table 2:** An overview of the excipients used in modified release semisolid ocular formulations (gels, hydrogels, and creams).

Release Rate *	API(s)	Excipients Used in the Formulation	Reference
prolonged	ibuprofen	Miglyol^®^ 812, lecithin, Kolliphor^®^ EL, glycerol	[71]
sustained	besifloxacin	CS, PVA, Gellan gum (GelriteTM)	[73]
prolonged/sustained	levofloxacin	CS, Tripolyphos-phate, sodium alginate, HPMC	[74]
sustained	levofloxacin	CS	[75]
prolonged/sustained	timolole malate	CS—gelatin crosslinked to β-CDs lysozyme, and β-glycerophosphate disodium salt hydrate, PBS, sodium fluorensic	[76]
sustained	latanoprost, curcumin	CS, PLGA, PVA	[77]
sustained	ketoconazole	Poly(*N*-isopropylacrylamide)/HA	[89]
prolonged	moxifloxacin hydrochloride	poloxamer F-127, gellan-gum, carbopol	[101]
sustained (8 h)	voriconazole	poloxamers P407, P188, CMC, BZC, NaCl	[102]
sustained	dexamethasone	poloxamer 407/188, Soy lecithine, glycerol, polycarbophil	[103]
prolonged	timolol maleate, dexamethasone, dorzolamide HCl	poloxamers P407, P188, CS gel	[104]
sustained	insuline	PLGA-PEG-PLGA CS, Sodium tripolyphosphate (TPP) (solution to form NPs)	[112]
prolonged (4–7 weeks)	rhodamine B, coumarin 6	PLGA-PEG-PLGA, PBS powder, Sulfo-cyanine 7 NHS ester (Cy7), cyanine 7.5 alkyne (Cy7.5)	[113]
extended (for 6 months)	ranibizumab	(PEG-PLLA-DA/NIPAAm) PLGA microspheres, PVA, Bovine serum albumin (BSA), PEG, Mg(OH)_2_	[152]
extended (for 6 months)	aflibercept	(PEG-PLLA-DA/NIPAAm) PLGA microspheres, PVA, Bovine serum albumin (BSA), PEG, Mg(OH)_2_	[153]
sustained	pilocarpine RGFP966	4-hydroxy-3,5-dimethoxybenzoic acid CS-g-poly(*N*-isopropylacrylamide)	[154]
sustained	dorzolamide-HCl	chol/Span 40 *L*-a-lecithin, chtiosan	[155]
sustained for 3 h	olopatadine HCl	gellan gum, carbopol 934P, benzododecenium bromide	[156]

* Release rate as stated by the author(s). Abbreviations: BZC: benzalkonium chloride, CKC: cetalkonium chloride, CS: chitosan, DMSO: hydrochloridedimethyl sulfoxide, HA: hyaluronic acid, PEG: polyethylene glycol, PG: propylene glycol, PVA: polyvinyl alcohol, HEC: hydroxyethylcellulose, PBS: phosphate buffered saline.

**Table 3:** An overview of the excipients used in modified release ocular formulations (inserts/films).

Release Rate *	API(s)	Excipients Used in the Formulation	Reference
sustained	fluorescein sodium salt	CS, poly(2-ethyl-2-oxazoline), PBS	[78]
maintance of mucoadhesiveness		CS, acetic acid	[79]
immediate (lev), sustained (dex)	dexamethazone, levofloxasin	HA, Glycol CS	[90]
sustained	acetazolamide	HA sodium salt, HP-β-CD, TEA, PEG, diglycidylether, 2,3-bis(2-methoxy-4-nitro-5-sulphophenyl)-2H-tetrazolium-5-carboxanilide inner salt	[91]
sustained	ketorolac tromethamine diclofenac sodium salt, vit E	CKC, sterylamine Dulbecco’s PBS, stearylamine, flur-biprofen sodium	[125]
graduated	chloramphenicol hemisuccinate, adrenaline	Gellan maleate/N-isopropylacrylamide Gellan gum => gelan maleate, BIS, TEMED/APS	[157]
sustained for more than 6 h	amlodipine	sulphobutyl-ether-β-CD, β-CD, hydro-xypropyl-β-CD	[158]
sustained	doxycyclin, glial-cell derived neurotrophic factor	alginate-collagen	[159]

* Release rate as stated by the author(s). Abbreviations: CKC: cetalkonium chloride, CS: chitosan, HA: hyaluronic acid, PEG: polyethylene glycol.

**Table 4:** An overview of the excipients used in modified release ocular formulations (nanotechnology).

Release Rate *	API(s)	Excipients Used in the Formulation	Reference
sustained	acetazolamide	poly-*γ*-glutamic acid—HA, PVA, soya bean lecithin, L-α-soya bean phosphatidyl choline, sodium hyaluronate, Leucine/Mannitol/PEG/glycerol, betamethasone sodium phosphate, betamethasone dipropionate ampoule, PEG 400	[142]
sustained	ibuprofen	soybean phospholipids, cholesterol, octadecylamine	[143]
prolonged	carvedilol	soy phosphatidylcholine, cetyltrimethylammonium/dimethyldidodecylammonium bromide	[92]
prolonged	moxifloxacin HCl	1,2-Dipalmitoyl-sn-glycero-3-phosphoethanolamine, cholesterol, egg phospholipid, CS, Tripolyphosphate sodium, 1-ethyl-3-(3-dimethylaminopropyl) carbodiimide hydrochloride, N-Hydroxysuccinimide	[144]
sustained	dorzolamide HCl	Leucaena leucocephala galactomannan (CMLLG/AMLLG)-NaOH	[160]

* Release rate as stated by the author(s). Abbreviations: CS: chitosan, HA: hyaluronic acid, PEG: polyethylene glycol, PVA: polyvinyl alcohol.

**Table 5:** Key characteristics of excipients and their potential advantages/disadvantages [161,162].

Excipients	Key Characteristics	Potential Advantages	Potential Disadvantages
chitosan	Deacetylation Molecular weight Solubility Contains hydroxyl and amino groups/polycationic in H+ env.	Interacts with negatively charged => delivery to mucosa Biocompatible Biodegradable Nontoxic Antimicrobial Antioxidant Limits growth of cancer cells	Final products of remarkable variability
hyaluronic acid	Anionic in physiological pH Has groups suitable for modification	Improves tissue hydration, lubricates Improves tissue resistance Widely available Fully resorbable Biocompatible Removes free radicals	Marginal side effects
poloxamer	Nonionic Amphiphilic Liquid/paste/flakes	Thermoreactive	Unstable from 20–25 °C Concentration needed to achieve gelation is high, affecting osmolarity
PLGA	Linear Soluble in various solvents Nontoxic	Biocompatible Highly crystalline (PLLA) Completely amorphous (PDLA) Can encapsulate molecules of virtually any size	
PVCL–PVA–PEG	Amphiphilic Soluble both in water and in organic solvents	Increases solubility of poorly soluble drugs/increases bioavailability through the formed micelles Does not absorb moisture from air	
cetalkonium chloride	Positively charged High lipophilicity Low toxicity	Antiseptic properties	
gelatin	High dissolution in water	Gelling emulsifying and foaming properties Widely available Not expensive Not as antigenic as collagen Groups can be chemically modified	None for long-term drug delivery systems Low rate of degradation
